# Association of Maxillary Labial Frenum Attachment and Insertion With Midline Diastema in Children With Primary Dentition: A Cross-Sectional Study

**DOI:** 10.7759/cureus.89892

**Published:** 2025-08-12

**Authors:** Zainab Khan, Akash Bhatnagar, Zakiya Perveen, Deepshikha Rajput, Palak Mishra, Ramakrishna Yeluri

**Affiliations:** 1 Department of Pedodontics and Preventive Dentistry, Teerthanker Mahaveer Dental College and Research Centre, Moradabad, IND; 2 Department of Pedodontics and Preventive Dentistry, Sharad Pawar Dental College and Hospital, Wardha, IND

**Keywords:** children, dentition, diastema, frenum, primary

## Abstract

Introduction: Midline diastema, a prevalent dental anomaly in young children, is often associated with labial frenum characteristics, including attachment type and insertion level. Despite its clinical significance, the relationship between these frenum features and midline diastema in primary dentition remains unexplored. This cross-sectional study aimed to investigate the prevalence of labial frenum variations and their impact on midline diastema in children aged 3-6 years, providing insights into their developmental and clinical implications.

Materials and methods: This cross-sectional study included 432 children aged 3-6 years with complete primary dentition and midline diastema. Maxillary labial frenum attachment types were categorized as mucosal, gingival, papillary, or papillary-penetrating. Frenal insertion levels were assessed as mid-attached gingiva, mucogingival junction, or inferior margin of the alveolar papillae. Intraoral examinations were performed by calibrated examiners using visual and tactile methods, with midline diastema measured to the nearest 0.1 mm and classified according to severity. Data were analyzed using descriptive statistics and chi-square tests for association. The Spearman correlation test was used to assess the correlation between frenum characteristics, age, and midline diastema.

Results: Maxillary frenum attachment and insertion levels showed significant age-dependent patterns, with gingival and mid-gingival insertions increasing with age, whereas papillary and alveolar papilla insertions decreased. Midline diastema was more prevalent and larger in younger children, decreased with age, and showed negative correlations with specific frenum attachments and insertion levels.

Conclusion: This study demonstrated that maxillary frenum attachment and insertion levels shifted with age, favoring gingival and mid-attached gingival insertions. These findings advocate for age-specific evaluations and a conservative approach to managing midline diastema in primary dentition.

## Introduction

The labial frenum, a mucosal fold containing connective tissue and occasionally muscle fibers, plays a crucial role in stabilizing the upper lip and anchoring it to the alveolar mucosa [1]. Its morphology and attachment type in primary dentition are critical for oral function, aesthetics, and dental alignment [2]. Variations in frenum morphology, such as thickness, length, and attachment level, have been implicated in the development of midline diastema, a gap between the maxillary central incisors [1]. While midline diastema is often considered a transient feature in primary dentition due to normal growth and development, persistent or pronounced diastema associated with aberrant frenulum characteristics may warrant clinical attention [2]. Midline diastema, gingival recession, interdental bone resorption, inadequate lip mobility, suboptimal oral hygiene practices, and dental misalignment are sequelae that may arise from inappropriate frenulum attachment [2,3]. Understanding the interplay between labial frenum characteristics and midline diastema in young children is essential for early diagnosis and intervention to prevent future orthodontic complications.

In primary dentition, the labial frenum undergoes significant changes as the child grows. Its attachment may shift apically with the eruption of permanent teeth; however, anomalies in its morphology or attachment type (e.g., gingival, papillary, or papillary-penetrating) can influence dental alignment and soft tissue health [2,4]. Previous studies have suggested that a low-attachment or thick labial frenum may contribute to midline diastema by exerting mechanical pressure or limiting lip movement, which can affect the positioning of maxillary incisors [1,2,5]. However, the exact relationship between frenum morphology, attachment type, and midline diastema in primary dentition remains unclear, particularly in diverse populations. Most existing research focuses on permanent or mixed dentition, leaving a significant gap in the understanding of these associations in younger children with primary teeth.

The clinical relevance of studying labial frenum characteristics in primary dentition lies in their potential to inform early orthodontic and pediatric dental care. If unresolved, midline diastema may lead to aesthetic concerns, functional issues such as improper speech articulation, or an increased risk of periodontal complications [2,6]. Early identification of frenum-related factors contributing to diastema can guide timely interventions, such as laser frenectomy or orthodontic monitoring, to mitigate long-term sequelae [7]. Moreover, the lack of standardized criteria for assessing frenum and its impact on diastema in the primary dentition underscores the need for systematic clinical evaluation. Existing literature often relies on subjective assessments or case reports, with few cross-sectional studies providing robust data on the prevalence and correlations in young populations.

This study addressed these gaps by conducting a cross-sectional evaluation of maxillary labial frenum attachment type and its association with midline diastema in children with primary dentition. This study aimed to investigate the relationship of different frenal attachments with midline diastema in a pediatric population. The objectives were to classify maxillary labial frenum attachment types in primary dentition using standardized criteria, and analyze the association between frenum characteristics and the presence or severity of midline diastema. By providing evidence-based insights into these relationships, this study aims to enhance clinical decision-making and contribute to the limited body of knowledge on frenum-related dental anomalies in young children.

This article was previously published as an abstract in the Journal of Indian Society of Pedodontics and Preventive Dentistry in February 2024 (DOI: 10.4103/jisppd.jisppd_59_24).

## Materials and methods

Study design and setting

This cross-sectional study was conducted at the Department of Pedodontics and Preventive Dentistry, Teerthanker Mahaveer Dental College and Research Centre, and the Department of Pediatrics, Teerthanker Mahaveer Medical College and Research Centre, Moradabad, Uttar Pradesh, India, from April 2022 to March 2024. This study adhered to the Strengthening the Reporting of Observational Studies in Epidemiology (STROBE) guidelines to ensure robust reporting standards. Ethical approval was obtained from the Institutional Ethics Committee of Teerthanker Mahaveer Dental College and Research Centre (Ref. No. TMDCRC/IEC/21-22/PPD4, dated 03/03/2022). Informed consent was obtained from the parents or legal guardians of all participating children, and assent was sought from children, where appropriate, ensuring compliance with ethical standards.

Sample size estimation

The sample size was calculated using an online statistical calculator (calculator.net) based on a population of 1500 children visiting the outpatient department over two years. With an estimated diastema prevalence of 30% [6], a 5% margin of error, and a 90% confidence level, the required sample size was 432 participants. This conservative proportion (p=0.5) maximized variability to ensure robustness. Therefore, 432 participants were enrolled in the primary dentition phase of the study.

Eligibility criteria

Children aged 3-6 years with complete eruption of the maxillary central incisors and midline diastema with normal complete primary dentition and no or minimal crowding were included. Participants were required to be free of any systemic diseases. The exclusion criteria included loss of maxillary anterior teeth, alterations in the shape or size of incisors, caries or restorations in the upper central incisors, history of interceptive or corrective orthodontic treatment, cleft lip abnormalities, orofacial syndromes, congenital deformities, and any surgeries or accidental injuries in the maxilla. These criteria ensured a homogeneous study population to accurately assess the relationship between labial frenum characteristics and midline diastemas.

Clinical examination

Intraoral examinations were performed by two trained and calibrated examiners using a mouth mirror and explorer under adequate illumination. The participants were seated upright on an ordinary or dental chair. The labial frenum was examined by gently lifting the upper lip using the index finger and thumb of both hands, following the direct visual method described by Mirko et al. [8]. Frenum attachment types were classified based on Mirko et al.’s classification [8], with permission obtained for its use (see Appendices). Midline diastema was measured by marking the midpoints of the mesial surfaces of the maxillary central incisors using a divider and ruler, with measurements recorded to the nearest 0.1 mm. All findings were documented in a standardized format, and intraoral photographs were taken for record-keeping and verification.

Classification of labial frenum

The labial frenum was classified using two established systems to evaluate its attachment and morphology. According to Mirko et al. [8], frenum attachment is categorized into four types: mucosal, where the frenum attaches to the mucogingival junction; gingival, where it attaches to the attached gingiva; papillary, where it attaches to the interdental papilla; and papillary-penetrating, where it extends into the palate beyond the interdental papilla. Additionally, the study of frenal insertion points is critical for understanding their anatomical and functional implications. Frenal attachments were categorized based on their position relative to the oral mucosa, including mid-attached gingiva, where the frenum was anchored centrally within the gingival tissue; the mucogingival junction, marking the transition between keratinized and non-keratinized mucosa; and the inferior margin at the alveolar papilla, where the frenum was inserted near the base of the interdental papilla. These insertion sites were assessed to determine their effects on periodontal health. These variations were assessed using visual and tactile evaluations. Midline diastema was recorded as present if the gap between the maxillary central incisors was ≥0.5 mm or absent if <0.5 mm, with further classification into mild (0.5-2 mm), moderate (2-4 mm), or severe (> 4 mm) based on the measured distance. Only cases with midline diastema were included.

Calibration of examiners

Both examiners underwent calibration training before the study to ensure consistency. Calibration involved assessing 30 children who were not included in the main study, with frenum attachment type classified independently by each examiner. Inter-examiner reliability was evaluated using Cohen’s kappa coefficient, achieving a kappa value ≥0.85, indicating excellent agreement. Intra-examiner reliability was assessed by re-examining 10% of the participants after a two-week interval using a kappa value of ≥0.90. Any discrepancies were resolved through discussion and consensus, guided by a senior pediatric dentist.

Statistical analysis

Data were entered into a Microsoft Excel (Microsoft Corp., Redmond, WA) spreadsheet and analyzed using Statistical Package for Social Sciences (SPSS) version 21 (IBM Corp., Armonk, NY). Descriptive statistics, including frequencies and proportions, were calculated for qualitative variables (frenum attachment type and presence of diastema). Quantitative variables, such as diastema width, were summarized using the mean and standard deviation. The normality of the data was checked using the Shapiro-Wilk test, and the data were found to be normally distributed. The chi-squared test was used to evaluate the associations between the categorical variables. Differences were considered statistically significant at p≤0.05, determining the relevance of the observed relationships. The Spearman correlation test was used to assess the correlations between frenum characteristics, age, and midline diastema.

## Results

The sample consisted of 216 (50%) males with a mean age of 5.21±2.02 years, and 216 (50%) females with a mean age of 4.43±2.45 years (Table 1).

**Table 1:** Descriptive statistics of the study sample Age is presented as the mean and standard deviation. Sex is presented as frequency (n) and percentage (%), where n denotes the number of participants.

Parameter	Unit	Male	Female	Overall
Age (in years)	Mean ± SD	5.21±2.02	4.43±2.45	4.82±2.25
Sex	n (%)	216 (50%)	216 (50%)	432 (100%)

The analysis revealed a statistically significant association between the type of frenal attachment and age (χ²=119.31, p=0.001). Gingival attachment showed a clear age-dependent increase. In contrast, mucosal attachment remained relatively stable across the age groups. Both papillary and papillary-penetrating attachments exhibited a marked decline with age. These findings suggest that gingival attachment becomes more prevalent with age, whereas papillary-related attachments diminish, possibly due to physiological maturation or periodontal changes. This strong statistical significance underscores the importance of age in frenal attachment patterns (Table 2).

**Table 2:** Association of type of frenal attachment with different age groups *p<0.05 denotes significance using the chi-square test of association. Data are presented as frequency (n) and percentage (%), where n denotes the number of participants.

Age groups	3-3.5 years		3.5-4 years		4-4.5 years		4.5-5 years		5-5.5 years		5.5-6 years		Chi statistics	p-value
Frenal attachment	n	%	n	%	n	%	n	%	n	%	n	%		
Gingival	12	2.8%	24	5.6%	33	7.6%	41	9.5%	46	10.7%	61	14.1%	119.31	0.001*
Mucosal	8	1.9%	9	2.1%	12	2.8%	13	3.0%	13	3.0%	11	2.6%		
Papillary	22	5.1%	16	3.7%	16	3.7%	10	2.3%	9	2.1%	0	0.0%		
Papillary-penetrating	30	6.9%	23	5.3%	11	2.6%	8	1.9%	4	0.9%	0	0.0%		

The analysis revealed a highly significant association between frenal insertion level and age group (χ²=141.41, p<0.001). Mid-attached gingival insertion demonstrated a progressive age-related increase. Conversely, insertions at the mucogingival junction remained relatively stable across age groups, whereas alveolar papilla insertions showed a marked decline with advancing age. These findings suggest a distinct maturation pattern in frenal insertion levels, where the attachment point appears to migrate apically with age. The near-complete disappearance of papillary insertions and the increasing prevalence of mid-attached gingival insertions in older groups may reflect normal periodontal development or age-related gingival changes. This strong statistical significance underscores the importance of considering age when evaluating frenal insertion patterns (Table 3).

**Table 3:** Association of point of insertion of frenum and age groups *p<0.05 denotes significance using the chi-square test of association. Data are presented as frequency (n) and percentage (%), where n denotes the number of participants.

Age groups	3-3.5 years		3.5-4 years		4-4.5 years		4.5-5 years		5-5.5 years		5.5-6 years		Chi statistics	p-value
Frenal insertion	n	%	n	%	n	%	n	%	n	%	n	%		
Mid-attached gingival	11	2.6%	23	5.3%	35	8.1%	45	10.4%	46	10.7%	72	16.7%	141.41	0.001*
Mucogingival junction	8	1.9%	10	2.3%	10	2.3%	9	2.1%	13	3.0%	0	0.0%		
Inferior margin at the alveolar papilla	53	12.3%	39	9.0%	27	6.3%	18	4.2%	13	3.0%	0	0.0%		

The analysis revealed a highly significant association between midline diastema and age (χ²=212.03; p<0.001). Smaller diastemas progressively increased with age, while larger diastemas (≥1.1 mm) were predominantly found in younger groups and were completely absent after 4.5 years of age. Moderate diastemas (0.5-1 mm) peaked in the middle-aged group before declining. These findings suggest the natural closure of larger diastemas with age, with smaller diastemas persisting or developing during dental maturation. The results indicated that diastema size was age-dependent (Table 4).

**Table 4:** Association of midline diastema with age groups *p<0.05 denotes significance using the chi-square test of association. Data are presented as frequency (n) and percentage (%), where n denotes the number of children.

Age groups	3-3.5 years		3.5-4 years		4-4.5 years		4.5-5 years		5-5.5 years		5.5-6 years		Chi statistics	p-value
Midline diastema	n	%	n	%	n	%	n	%	n	%	n	%		
<0.5 mm	15	3.5%	10	2.3%	34	7.9%	38	8.8%	44	10.2%	48	11.1%	212.03	0.001*
0.5 to 1 mm	15	3.5%	22	5.1%	32	7.4%	34	7.9%	28	6.5%	24	5.6%		
1.1 to 1.5 mm	15	3.5%	12	2.8%	4	0.9%	0	0.0%	0	0.0%	0	0.0%		
1.6 to 2 mm	21	4.9%	28	6.5%	2	0.5%	0	0.0%	0	0.0%	0	0.0%		
>2 mm	6	1.4%	0	0.0%	0	0.0%	0	0.0%	0	0.0%	0	0.0%		

Correlation analysis revealed significant relationships between the key variables (p<0.05). Age showed moderate positive correlations with frenal attachments (r=0.38) and frenal insertion (r=0.38), but a strong negative correlation with midline diastema (r=-0.51). Midline diastema was significantly negatively correlated with age, frenal attachment, and frenal insertion. This implies that younger individuals are more likely to exhibit midline diastema, potentially due to developmental factors or less mature periodontal structures. Additionally, certain frenal insertion points may exert mechanical influences that either restrict or limit diastema persistence (Table 5).

**Table 5:** Assessment of correlation between outcome variables *p<0.05 denotes statistical significance using the Spearman correlation test. Very weak correlation: 0.0<∣r∣<0.20; weak: 0.2≤∣r∣<0.4; moderate: 0.4≤∣r∣<0.6; strong: 0.6≤∣r∣<0.8; and very strong: 0.8≤∣r∣≤1.

Variables	r-value/p-value	Age groups	Frenal attachments	Frenal insertion	Midline diastema
Age groups	r-value	1	0.38	0.38	-0.51
	p-value	0.999	0.001*	0.001*	0.001*
Frenal attachments	r-value	0.38	1	0.91	-0.59
	p-value	0.001*	0.999	0.001*	0.001*
Frenal insertion	r-value	0.38	0.91	1	-0.62
	p-value	0.001*	0.001*	0.999	0.001*
Midline diastema	r-value	-0.51	-0.59	-0.62	1
	p-value	0.001*	0.001*	0.001*	0.999

## Discussion

This lack of significant age-related differences noted in the present study may be attributed to the relatively short age range (3-6 years) and primary dentition phase, where developmental changes in frenum morphology are minimal. In contrast, Mirko et al. [8] suggested that frenum insertion types may evolve with dental eruption stages, particularly as children transition to mixed dentition, where mechanical forces from erupting permanent teeth could alter the frenum appearance. The absence of such a transition in the study population likely explains this non-significant association.

A highly significant association was observed between frenal attachment type and age. The increase in gingival attachment and decline in papillary and papillary-penetrating attachments with age corroborate the findings of previous studies [9,10]. The stability of mucosal attachments across age groups suggests that this attachment type is less influenced by age-related changes in the primary dentition. However, the marked decline in papillary attachments could be linked to the physiological remodeling of the interdental papilla as children age, which is potentially driven by gingival tissue maturation or changes in occlusal dynamics. The strong statistical significance underscores the importance of age as a determinant of frenal attachment, suggesting that clinicians should consider age-specific patterns when assessing frenum-related issues in pediatric patients. A prior investigation indicated a minimal increase in the vertical dentoalveolar segment of the maxilla, resulting in a marginal vertical alteration in the insertion of the maxillary frenum [11]. Any disruption in the repositioning of the continuous band of connective tissue associated with the frenum during alveolar process development may contribute to the emergence of midline diastema and concurrently influence the growth of the anterior maxilla [12]. In such cases, the frenum may persist in its primordial condition, indicating developmental stagnation. Díaz-Pizan et al. [5] reported an inverse relationship between frenal insertion level and midline diastema, consistent with the present study.

The highly significant association between the frenal insertion level and age highlights a clear pattern of insertion point migration. The progressive increase in mid-attached gingiva insertion and the decline in alveolar papilla insertion with age align with the natural apical shift of frenal attachments described by Boutsi and Tatakis [13]. This shift may reflect periodontal development, where the gingival tissue thickens and the attachment point moves apically owing to changes in the mucogingival complex. The stability of mucogingival junction insertions across age groups suggests that this site is less affected by developmental changes in the primary dentition. In contrast, Sewerin [14] noted that alveolar papilla insertions may persist in cases of abnormal frenum morphology, such as bifid or wider frena, which could explain their presence in younger age groups in the present study sample. The near-complete disappearance of papillary insertions in the older groups supports the hypothesis that frenal insertion points adapt to the changing periodontal environment, potentially influenced by factors such as tooth eruption and gingival remodeling.

The highly significant negative correlation between midline diastema and age groups indicates that larger diastemas are more prevalent in younger children and diminish with age, whereas smaller diastemas persist or develop in middle-aged groups [15]. This is consistent with the natural closure of midline diastema during primary dentition, as reported in previous studies [5,15], which attributed this phenomenon to the eruption of adjacent teeth and physiological spacing adjustments. The complete absence of larger diastemas in older age groups (beyond the age of 3 years) suggests a developmental closure mechanism, possibly driven by increased masticatory forces or periodontal stabilization.

Diastemas measuring ≤2 mm are likely to resolve in the absence of a deep bite, concurrent with the eruption of permanent canines. Conversely, in certain cases, a diastema measuring 3 mm or greater may necessitate orthodontic intervention for closure, using either removable or fixed appliances before the eruption of canines [16]. A notable increase in the prevalence of mucosal and gingival frenum variations was observed in a cohort of adult individuals with a minor diastema of ≤2 mm, whereas a larger diastema exceeding 2 mm was correlated with papillary and papillary-penetrating frenum types [17].

The significant negative correlations between midline diastema, frenal attachments, and frenal insertion suggest that specific attachment types and insertion points influence the presence or severity of the diastema. For instance, gingival and mid-attached gingival insertions, which increase with age, may exert stabilizing forces on the maxillary central incisors, thereby reducing diastema size. A previous study reported a lower incidence of diastema in cases with gingival frenal insertion [17]. Conversely, papillary or alveolar papilla insertions may contribute to diastema persistence by mechanically separating incisors [17]. These findings are consistent with those of Mirko et al. [8]. Diastema is notably correlated with papillary and papillary-penetrating frenum types, as indicated in several studies [4,11,17].

Clinical implications

These findings have significant clinical implications for pediatric dentistry. Age-dependent patterns of frenal attachment and insertion suggest that clinicians should tailor their assessments based on a child’s age and developmental stage. For instance, younger children with papillary or alveolar papilla insertions may require closer monitoring for midline diastema, as these attachments are associated with larger gaps. The natural closure of diastemas with age supports a conservative approach to treatment in primary dentition, avoiding unnecessary interventions unless the diastema persists beyond age six or is associated with pathological frenum characteristics. These findings emphasize the importance of early screening to identify high-risk frenal features that may warrant future orthodontic or surgical intervention.

Future recommendations

Future research should explore longitudinal changes in frenum characteristics and midline diastema during transition from primary to mixed dentition, as this could clarify the persistence of diastema and the evolution of frenulum attachments. Larger multicenter studies involving diverse populations would enhance generalizability, as regional differences in periodontal anatomy may exist among different populations. Additionally, investigating the biomechanical properties of different frenum types and their impact on tooth movement could provide deeper insights into the causal mechanisms. Incorporating advanced imaging techniques, such as 3D intraoral scanning, could improve the precision of frenum and diastema measurements and address the potential limitations of manual methods.

Limitations

The cross-sectional design of this study limits its ability to track changes over time, which could provide stronger evidence of causal relationships. The sample was restricted to a specific geographic region, potentially limiting the generalizability of the results owing to genetic or environmental variations. Reliance on visual and tactile examinations, despite high inter-examiner reliability, may introduce minor measurement errors compared to advanced imaging techniques. Additionally, the exclusion of children with systemic diseases or congenital deformities may have overlooked potential confounding factors affecting the frenum and diastema development.

## Conclusions

This study found significant associations between age and both frenal attachment and insertion levels, showing a shift toward gingival and mid-attached gingival insertions, with a decline in papillary and alveolar papilla insertions with increasing age. Midline diastema showed a strong negative correlation with age, frenal attachments, and insertion levels, indicating that larger diastemas are more prevalent in younger children and tend to diminish with age, likely due to specific frenal characteristics. These findings highlight the importance of age and frenal features in assessing midline diastema in children with primary dentition, supporting a conservative management approach and the need for age-specific clinical evaluations.

## Figures and Tables

**Figure 1:**
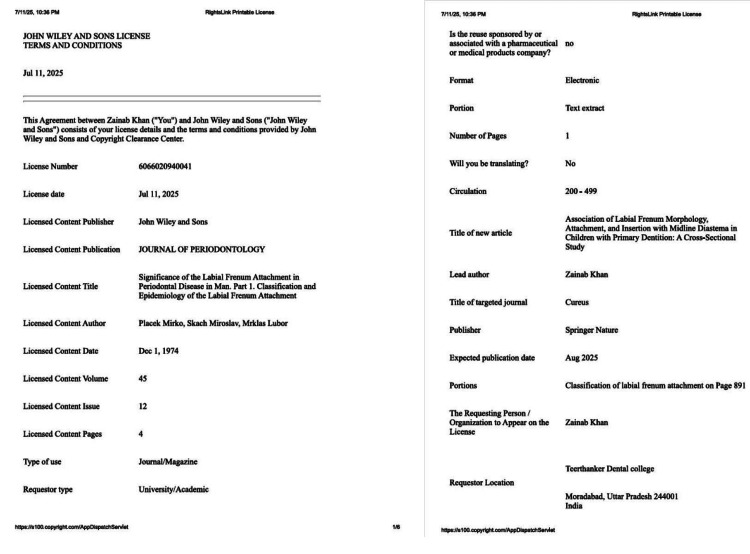
Permission letter from John Wiley and Sons, dated July 11, 2025, granting use of the labial frenum attachment classification by Mirko et al. (1974) published in the Journal of Periodontology.

## References

[1] Kinney R, Burris RC, Moffat R, Almpani K (2024). Assessment and management of maxillary labial frenum-a scoping review. Diagnostics (Basel).

[2] Biradar SM, Patil AY, Kotnoor SS, Bacha S, Bijjaragi SC, Kattimani PT (2020). Assessment of diverse frenal morphology in primary, mixed, and permanent dentition: a prevalence study. J Contemp Dent Pract.

[3] Patil RS, Rahate I, Fulzele P, Dubey R, Yeluri R, Solanki D (2024). Pediatric high labial frenum management: utilizing a diode laser for the transformation of the smile of an eight-year-old child. Cureus.

[4] Jindal V, Kaur R, Goel A, Mahajan A, Mahajan N, Mahajan A (2016). Variations in the frenal morphology in the diverse population: a clinical study. J Indian Soc Periodontol.

[5] Díaz-Pizán ME, Lagravère MO, Villena R (2006). Midline diastema and frenum morphology in the primary dentition. J Dent Child (Chic).

[6] Jonathan PT, Thakur H, Galhotra A, Galhotra V, Gupta N (2018). Maxillary labial frenum morphology and midline diastema among 3 to 12-year-old schoolgoing children in Sri Ganganagar city: a cross-sectional study. J Indian Soc Pedod Prev Dent.

[7] Delli K, Livas C, Sculean A, Katsaros C, Bornstein MM (2013). Facts and myths regarding the maxillary midline frenum and its treatment: a systematic review of the literature. Quintessence Int.

[8] Mirko P, Miroslav S, Lubor M (1974). Significance of the labial frenum attachment in periodontal disease in man. Part I. Classification and epidemiology of the labial frenum attachment. J Periodontol.

[9] Townsend JA, Brannon RB, Cheramie T, Hagan J (2013). Prevalence and variations of the median maxillary labial frenum in children, adolescents, and adults in a diverse population. Gen Dent.

[10] Rajani ER, Biswas PP, Emmatty R (2018). Prevalence of variations in morphology and attachment of maxillary labial frenum in various skeletal patterns - a cross-sectional study. J Indian Soc Periodontol.

[11] Schuepbach I, Vento C, Denes BJ, Antonarakis GS, Kiliaridis S (2023). Longitudinal changes of the insertion of the maxillary labial frenum in children and adolescents undergoing orthodontic treatment. Am J Orthod Dentofacial Orthop.

[12] Priyanka M, Sruthi R, Ramakrishnan T, Emmadi P, Ambalavanan N (2013). An overview of frenal attachments. J Indian Soc Periodontol.

[13] Boutsi EA, Tatakis DN (2011). Maxillary labial frenum attachment in children. Int J Paediatr Dent.

[14] Sewerin I (1971). Prevalence of variations and anomalies of the upper labial frenum. Acta Odontol Scand.

[15] Edwards JG (1977). The diastema, the frenum, the frenectomy: a clinical study. Am J Orthod.

[16] Huang WJ, Creath CJ (1995). The midline diastema: a review of its etiology and treatment. Pediatr Dent.

[17] Sękowska A, Chałas R (2017). Diastema size and type of upper lip midline frenulum attachment. Folia Morphol (Warsz).

